# Macrophage Activation Syndrome, Glomerulonephritis, Pericarditis, and Retinal Vasculitis as Initial Presentation of Systemic Lupus Erythematosus

**DOI:** 10.1155/2018/5979386

**Published:** 2018-09-26

**Authors:** Yiming Luo, Yumeng Wen, Ana Belen Arevalo Molina, Punya Dahal, Lorenz Leuprecht, Makda Bsrat

**Affiliations:** Department of Medicine, Mount Sinai St Luke's and Mount Sinai West, Icahn School of Medicine at Mount Sinai, New York, NY, USA

## Abstract

Macrophage activation syndrome (MAS) is a rare manifestation of systemic lupus erythematosus (SLE) with potentially life-threatening consequences. To the best of our knowledge, this is the first case reported in literature for a constellation of MAS, glomerulonephritis, pericarditis, and retinal vasculitis as initial presentation of SLE. Despite extensive multisystem involvement of his disease, the patient responded well to initial steroid treatment, with mycophenolate mofetil successfully added as a steroid-sparing agent. Our case highlights the importance of multispecialty collaboration in the diagnosis and management of SLE with multisystem involvement.

## 1. Introduction

Systemic lupus erythematosus (SLE) is a systemic autoimmune disorder which can involve almost all vital organs, including brain, eyes, heart, blood, and kidney [1]. The clinical heterogeneity of the disease presents a consistent challenge to clinicians. The reported prevalence of systemic lupus erythematosus (SLE) in the United States is 20 to 150 cases per 100,000, and the risk is higher among Asians, African Americans, African Caribbean, and Hispanic Americans compared with Caucasian [2].

Macrophage activation syndrome (MAS), also known as hemophagocytic lymphohistiocytosis (HLH), is a rare and potentially fatal complication of several autoimmune diseases, including juvenile idiopathic arthritis and SLE. The incidence of MAS associated with SLE is about 0.9–4.6% [3]. Excessive T-cell and macrophage activation and proliferation leads to hypercytokinemia and hemophagocytosis and can result in overwhelming multisystem inflammatory response [4].

We report a rare case of a combination of MAS, glomerulonephritis, pericarditis, and retinal vasculitis as the initial presentation of systemic lupus erythematosus. Despite the extensive involvement of his initial disease, the patient responded well to initial steroid treatment followed with mycophenolate mofetil.

## 2. Case Presentation

A 37-year-old Caucasian male with a past medical history of alcohol abuse was referred for inpatient admission after being found to have pancytopenia. He had subjective fever and productive cough with yellow sputum a few weeks ago, which resolved after a few days without treatment. He visited his primary care physician and laboratory exam showed pancytopenia, thus leading to the referral. He also endorsed generalized weakness, progressive right eye blurry vision, and chest tightness for the past few weeks. There was no skin rash, joint pain, hair loss, heartburn, and Raynaud's phenomenon. Physical exam was only remarkable for cervical nontender lymphadenopathy.

Upon admission, he was febrile with a maximum temperature of 101.2 F. Laboratory exam was notable for white blood cell 1,900 counts/*μ*L, hemoglobin 7.2 g/dL, hematocrit 21.2%, platelet 19,000 counts/*μ*L, sodium 126 mmol/L, and creatinine 1.44 mg/dL. Urinalysis showed elevated white blood cells, dysmorphic red blood cells, and proteinuria. Further work up showed that he had elevated ferritin 6542 ng/mL, high triglycerides 327 mg/dL, marked decreased complement levels (C3 14 mg/dL and C4 3 mg/dL), elevated ESR 137 mm/hr, strongly positive for antinuclear antibody (ANA, 1 : 640), and anti-double-stranded DNA antibody (anti-dsDNA, >1 : 1280). Anticardiolipin antibody (IgM and IgG) and beta-2 glycoprotein I antibody (IgG) were also positive. Direct antiglobulin test (Coomb's) was positive. Urine studies showed microalbumin to creatinine ratio of 1958 mg/g and protein creatinine ratio of 7.04 mg/mg consistent with nephrotic-range proteinuria. Transthoracic echocardiogram showed normal ventricular function but moderate circumferential pericardial effusion. Abdominal ultrasonography showed hepatosplenomegaly with evidence of cirrhosis. Based on the above findings, he was diagnosed with systemic lupus erythematosus, pericarditis, and lupus nephritis. Pulse steroid with methylprednisolone 1000 mg daily was started for 3 days, followed by oral prednisone 60 mg daily. His fever and chest pain resolved, and his blood cell counts and creatinine improved after the treatment. He subsequently underwent bone marrow biopsy which showed increased histiocytes with focal evidence of hemophagocytic cells consistent with macrophage activation syndrome considering his clinical presentation. Renal biopsy was also performed and confirmed diffuse proliferative and membranous lupus nephritis (Class IV/V) along with focal segmental glomerulosclerosis NOS type. He also underwent ophthalmology evaluation, and funduscopic exam with fluorescein angiogram showed cotton-wool spots with retinal hemorrhage consistent with retinal vasculitis. His laboratory examination continued to improve, and he was subsequently discharged from hospital one week after admission. Mycophenolate mofetil was started in addition to steroid upon discharge. Repeat transthoracic echocardiogram three weeks after treatment showed marked decrease in pericardial effusions. His disease remained stable while on mycophenolate mofetil with prednisone gradually tapered from 60 mg to 10 mg twelfth week after discharge. His vision acuity remained stable, and repeat funduscopic examination showed improvement of his retinal lesions. Detailed laboratory examination in the hospital and on follow-up visit is presented in Table 1.

## 3. Discussion

We present a case of an adult male SLE patient with multisystem involvement as initial presentation, including two rare and serious complications, MAS, and retinal vasculitis. The diagnosis of SLE was confirmed by the presence of lupus nephritis on biopsy together with typical biochemical finding, including positive ANA, anti-dsDNA, antiphospholipid antibodies, and low complement levels. Although a concomitant alcoholic hepatitis is essentially difficult to exclude given his known history of alcohol abuse, his hepatosplenomegaly is more likely from his underlying hematoimmunological response given his overall clinical picture. And his grossly normal liver transaminase and bilirubin also make alcoholic hepatitis highly unlikely.

The diagnosis of MAS in SLE can be challenging, as the presentation can mimic the clinical features of SLE or an infectious complication. One recent study analyzed 89 adult SLE patients with MAS and found that MAS was the initial presentation of SLE in 46% [5], thus highlighting the potentially crucial role of general internists or hospitalists as they may first encounter such patients before rheumatologists being involved. Hemophagocytosis is a late feature of HLH, and a negative bone marrow biopsy does not exclude the diagnosis of HLH/MAS [6]. Specific diagnostic criteria for MAS in SLE are only proposed in pediatric population [7], but not in adult population. The well-accepted HLH 2004 diagnostic criteria [8] and the proposed 2009 diagnostic criteria [9] can be used, but they were primarily developed for primary HLH. To aid the diagnosis of secondary HLH in adult population, Fardet et al. developed the HScore [10], whose performance in adult SLE population is unknown. Gavand recently analyzed 103 MAS episodes in 89 SLE adult populations and found that hyperferritinemia >500 *µ*g/L had the best sensitivity (96.2%) and the strongest indicator to separate MAS from active SLE [5]. Interestingly, the study also demonstrated significantly elevated procalcitonin in MAS episodes and could be considered as “red flag” for early diagnosis in adult SLE patients without concomitant infections [5]. Our patient fits into both HLH 2004 and 2009 diagnostic criteria (fever, hepatosplenomegaly, pancytopenia, hyperferritinemia, hypertriglyceridemia, hyponatremia, and presence of hemophagocytosis on bone marrow biopsy) and also with an HScore of 253, which translates into a 99.5% probability of having HLH/MAS.

The pathogenesis of MAS is not completely understood. The hallmark of the syndrome is characterized by excessive activation and proliferation of macrophage and T-lymphocytes, with massive release of proinflammatory cytokines [11]. Shimizu et al. [12] revealed a unique cytokine profile of SLE-MAS compared to systemic JIA-associated MAS and EBV-induced HLH, with a pattern of a TNF-*α* dominant increase and IgM-type antilymphocyte antibody detected on the surface of lymphocytes during the acute phase and disappeared upon remission. Recent studies also suggested that increased serum-free IL-18 levels are causatively involved in the MAS development [13].

There is no randomized controlled trial-based guideline for managing MAS in patients with SLE. Steroid is the cornerstone of treatment [14]. Second immunosuppressive can be added in severe or refractory cases, including cyclophosphamide [15], mycophenolate mofetil [16], rituximab [17], infliximab [18], anakinra [19], intravenous immunoglobulin [20], and plasma exchange [21]. HLH-2004 treatment protocol with dexamethasone, cyclosporine, and etoposide can also be considered in selected cases [4]. Although MAS is considered a lethal complication of SLE, recent studies showed that MAS in adult SLE population carried a better prognosis with mortality below 5% compared to other secondary HLH in historic cohort, and steroid alone was sufficient in majority of the cases [5].

Retinal vasculitis in SLE, or lupus retinopathy, has been found in 3 to 29% of patients with SLE. It often presents as bilateral visual loss, correlates with SLE disease activity, and is commonly associated with nephritis, central nervous system involvement, and antiphospholipid antibodies [22]. Typical findings of lupus retinopathy in the fluorescein angiography are tortuous retinal vessels, edema of papilla, hemorrhagic, or cotton-wool spots. One recent study has shown decreased survival in SLE patients with retinopathy compared to those without retinopathy, thus highlighting the importance of retinal diseases being both visually and prognostically [23]. The treatment of lupus retinopathy depends on the severity of disease, as visual outcome was usually better in those with cotton-wool spots than severe retinal vaso-occlusive disease [24]. Bevacizumab should be considered in severe vaso-occlusive retinopathy. In addition, vitrectomy and retinal photocoagulation can be performed in selected cases to halt neovascularization and prevent aggravation of visual loss [24]. Our patient's retinal disease did not show evidence of severe vaso-occlusive changes and responded well to the initial immunosuppressive treatment without the need for advanced treatment.

McCann et al. [21] reported a SLE case with similar initial presentations to ours: a pediatric female SLE patient presenting with MAS, retinal vasculitis, glomerulonephritis, and pancreatitis. That patient responded poorly with initial steroid treatment and subsequently required cyclophosphamide and plasma exchange [21]. On the contrary, with similar extensive multisystem involvement, our patient responded well to the initial steroid induction therapy without concurrent second immunosuppressive agent. And mycophenolate mofetil was subsequently added and successfully served as a steroid-sparing agent for his case. Apart from age as a possible prognostic factor [5], whether other underlying factors are present leading to such strikingly different response to treatment in these two rare cases with similar presentations deserve further investigations.

## 4. Conclusion

We presented a case of an adult male SLE patient initially presenting with multisystem involvement with a unique combination of MAS, glomerulonephritis, pericarditis, and retinal vasculitis. The patient responded well with the initial steroid induction therapy, and his disease remained stable with mycophenolate mofetil as a steroid-sparing agent. Our case highlights the unique feature of MAS in adult SLE population and also the importance of multispecialty collaboration in the diagnosis and management of SLE with multisystem involvement.

## Figures and Tables

**Table 1:** Laboratory examination in the hospital and during follow-up visit.

	Admission	Discharge	6 weeks after discharge	12 weeks after discharge
*Blood count*				
Leukocyte (K/*μ*L)	**1.9**	5.2	9.9	5.9
Hemoglobin (g/dL)	**6.5**	8	8.3	10.9
Hematocrit	**19.2**	23.7	26.3	32.5
Platelets (K/*μ*L)	**14**	**38**	**130**	236

*Chemistry*				
Sodium (mmol/L)	**127**	135	135	141
Potassium (mmol/L)	4.6	5.2	4.1	4.2
Chloride (mEq/L)	104	109	106	106
Bicarbonate (mmol/L)	21	22	22	
Blood urea nitrogen (mg/mL)	23	43	29	16
Creatinine (mg/mL)	**1.42**	**1.11**	**1.03**	**1.05**
Glomerular filtration rate	56	75	81	79
Calcium (mg/mL)	5.8	7	7.6	8.4
Glucose (mg/mL)	100	88	130	76
Aspartate aminotransferase (U/L)	19		17	18
Alanine aminotransferase (U/L)	33		21	10
Alkaline phosphatase (U/L)	47		53	44
Total bilirubin (mg/dL)	0.2		0.2	0.2
Total protein (g/dL)	4.4	4.8	4.3	4.6
Albumin (g/dL)	1.4	1.5	2.3	2.4
Ferritin (ng/mL)	**6542**	**1027**	**527**	330
Lactate dehydrogenase (U/L)	**336**	**252**	**275**	187
Haptoglobin (mg/dL)	**<8**	**20**	**<8**	52
Thyroid-stimulating hormone (uIU/mL)	7.5			
Creatine kinase (U/L)	105			
C-reactive protein (mg/L)	0.67			
Vitamin B12 (pg/mL)	414			
Triglycerides	327			

*Coagulation*				
Activated partial thromboplastin time (seconds)	31.5			
Prothrombin time (seconds)	14.2			
International normalized ratio	1.1			
Dilute Russell's viper venom time (seconds)	40.5			

*Others*				
Erythrocyte sedimentation rate (mm/hr)	**137**			
Complement 3 (mg/dL)	**14**			
Complement 4 (mg/dL)	**3**			
Direct antiglobulin test, broad-spectrum Coomb's serum	*Positive*			
Direct antiglobulin test, anti-IgG Coomb's serum	*Positive*			
Direct antiglobulin test, anti-C3 Coomb's serum	Negative			
Antibody identification	*Cold autoantibody*			

*Urine study*				
Protein	**3+**	**3+**	**1+**	**1+**
Protein/creatinine ratio	**7.04**			
Blood	**3+**	**3+**	**2+**	**2+**
Red blood cell (/HPF)	**60**		11–25	11–25
White blood cell (/HPF)	**7**		0–4	0–4

*Autoimmune serology*				
Antinuclear antibody	**1 : 640 ** *(homogeneous pattern)*			
Anti-dsDNA antibody (IU/ml)	**195**			
Anti-Ro antibody (AU/mL)	12			
Anti-ribonucleoprotein antibody (AI)	0.5			
Anti-Smith antibody (AI)	0.4			
Anti-Scl 70 antibody (AI)	0.2			
Rheumatoid factor (IU/mL)	<15			
Anti-cyclic citrullinated peptide antibody (units)	19			
Anticardiolipin antibody, IgM (U/mL)	**22**			
Anticardiolipin antibody, IgG (U/mL)	**48**			
Anticardiolipin antibody, IgA (U/mL)	<9			
Beta-2 glycoprotein I antibody, IgM (GPI IgM units)	<9			
Beta-2 glycoprotein I antibody, IgG (GPI IgG units)	**55**			
Beta-2 glycoprotein I antibody, IgA (GPI IgA units)	9			
